# Does Geography Play a Role in the Receipt of End-of-Life Care for Advanced Cancer Patients? Evidence from an Australian Local Health District Population-Based Study

**DOI:** 10.1089/jpm.2022.0555

**Published:** 2023-11-08

**Authors:** Jessica Cerni, Hassan Hosseinzadeh, Judy Mullan, Victoria Westley-Wise, Lorraine Chantrill, Greg Barclay, Joel Rhee

**Affiliations:** ^1^ Faculty of Arts, Social Sciences, and Humanities, School of Health and Society, University of Wollongong, Wollongong, New South Wales, Australia.; ^2^ Centre for Health Research Illawarra Shoalhaven Population (CHRISP), Graduate School of Medicine, University of Wollongong, Wollongong, New South Wales, Australia.; ^3^ Centre for Health Research Illawarra Shoalhaven Population (CHRISP), Illawarra Shoalhaven Local Health District (ISLHD), University of Wollongong, Wollongong, New South Wales, Australia.; ^4^ Department of Medical Oncology and Illawarra Shoalhaven Local Health District, Wollongong, New South Wales, Australia.; ^5^ Department of Palliative Care, Illawarra Shoalhaven Local Health District, Wollongong, New South Wales, Australia.; ^6^ School of Population Health, University of New South Wales, Sydney, New South Wales, Australia.; ^7^ Graduate School of Medicine, University of Wollongong, Wollongong, New South Wales, Australia.

**Keywords:** cancer, end of life, geography, health services research, palliative care, rural, travel time

## Abstract

**Objectives:**

To assess the influence of geographic remoteness on health care utilization at end of life (EOL) by people with advanced cancer in a geographically diverse Australian local health district, using two objective measures of rurality and travel-time estimations to health care facilities.

**Methods:**

This retrospective cohort study examined the association between rurality (using the Modified Monash Model) and travel-time estimation, and demographic and clinical factors, with the receipt of >1 inpatient and outpatient health service in the last year of life in multivariate models. The study cohort comprised of 3546 patients with cancer, aged ≥18 years, who died in a public hospital between 2015 and 2019.

**Results:**

Compared with decedents from metropolitan areas, decedents from some rural areas had higher rates of emergency department visits (small rural towns: aRR 1.29, 95% CI: 1.07–1.57) and ICU admissions (large rural towns: aRR 1.32, 95% CI: 1.03–1.69), but lower rates of acute hospital admissions (large rural towns: aRR 0.83, 95% CI: 0.76–0.90), inpatient palliative care (PC) (regional centers: aRR 0.85, 95% CI: 0.75–0.97), and inpatient radiotherapy (lowest in small rural towns: aRR 0.07, 95% CI: 0.03–0.18). Decedents from rural and regional centers had lower rates of outpatient chemotherapy and radiotherapy use, yet higher rates of outpatient cancer service utilization (*p* < 0.05). Shorter travel times (10–<30 minutes) were associated with higher rates of inpatient specialist PC (aRR 1.48, 95% CI: 1.09–1.98).

**Conclusions:**

Reporting on a series of inpatient and outpatient services used in the last year of life, measures of rurality and travel-time estimates can be useful tools to estimate geographic variation in EOL cancer care provision, with significant gaps uncovered in inpatient PC and outpatient service utilization in rural areas. Policies aimed at redistributing EOL resources in rural and regional communities to reduce travel times to health care facilities could help to reduce regional disparities and ensure equitable access to EOL care services.

## Introduction

Quality of end-of-life (EOL) care continues to gain attention as a key facet of excellence in cancer care,^1^ as does the disparities in EOL care service access among advanced cancer patients residing in rural and remote areas.^2^ Despite timely EOL care with acute and palliative intent having direct benefits,^3^ many patients with advanced cancer, who reside outside of metropolitan cities, continue to be underserved.^4^

Ensuring equitable access to quality EOL care across diverse populations has become a policy priority for both high- and low-income countries.^5^ Rural and remote populations often face large inequalities in the access and utilization of health services, with the provision of EOL care no exception. In Australia, where one-third of the population live outside major metropolitan centers,^6^ studies have revealed that the health care system still produces EOL care that is often fragmented and varied dependent upon a patient's geographic location. With policies such as the NSW Health EOL and Palliative Care Framework 2019–2024 recognizing the need to address urban–rural inequities of EOL care access, more research is needed on appropriate methods for measuring geographic access and to understand the root causes of these geographic disparities.

In rural settings, geographic access remains a key barrier,^2^ with many organizational and individual-level factors impeding the accessibility of quality EOL care.^7^ Rurality has been widely acknowledged in health policy making as a proxy for inaccessibility and often measured by geographic characteristics of an area (e.g., low population density or distance from health care resources).^8^ Distance is another measure of geographic access, one which presents individual barriers of cost, time, and inconvenience.^9^ Straight line distance or network distances to health care facilities including travel-time estimates are measures with added complexity but are often more relevant to health care utilization.^10^

International studies have previously referenced rurality and the inequity issues of EOL care access, although with high degrees of variation. For instance, studies from the United States and Canada found that rural cancer decedents had higher rates of emergency department (ED) visits in their last 30 days^11^ and six months^12^ of life compared with urban decedents. Similar results were found for increased hospital admissions among rural U.S.,^11,13^ Canadian,^13^ and German^14^ advanced cancer patient cohorts, as compared with their metropolitan counterparts.

In contrast, studies in the United States have shown an inverse relationship with a distinct underutilization of palliative care services such as hospice care in the last 12 months of life among rural patients.^14–16^ Despite rurality being a widely used measure in health research, no international consensus on the definition exists, often leading to the application of arbitrary measurements.

Increasing distance to services^17^ including travel time to hospitals and road network factors has also been associated with poor health outcomes.^18^ This is particularly important not only in developed countries such as Australia where the population is thinly spread over vast geography, but also in low-income countries where health services are scarce. With few studies finding lower rates of palliative radiotherapy,^19^ chemotherapy,^20^ and specialist palliative care (SPC)^21^ used among advanced cancer patients who experienced longer travel times to their nearest health care facility, the full extent of the rural–urban health differential in EOL cancer care in different settings remains unexamined.

More studies are needed, especially as rural patients often consider distance to health care facilities when deciding if and when to seek care.^9^ To date, limited studies have looked at distance in terms of travel time compared with commonly used rurality indexes as a determinant in EOL cancer care and of those who have, significant variation remains in the methods used. The aim of this study, therefore, was to assess the influence of geographic remoteness on EOL cancer care service utilization in an Australian local health district (LHD) by including two objective measures of rurality and travel-time estimations.

## Methods

### Study design and setting

A retrospective population-based cohort study was conducted using longitudinal administrative health data from an Australian LHD, which includes rural, regional, and metropolitan areas.^22^ Spread across an area of >50,000 km^2^, eight public hospital sites across three main hubs service >400,000 residents, five of which have an ED facility, two with an intensive care unit (ICU), two with radiotherapy services, and three with chemotherapy services. Three SPC units provide inpatient, consulting, and community SPC services to all residents of the LHD.

### Data sources and measures

Data on all decedents who died in a LHD hospital between January 1, 2015 and December 31, 2019 were obtained from the Illawarra Health Information Platform (IHIP), a nonidentifiable health records databank.^22^ Data included in this study were linked to routinely collected admitted patients data, sub- and nonacute inpatients data, ED data, nonadmitted patients data (NAP), and death audit data (DA). Data variables included sociodemographic, clinical, and geographic information.

**FIG. 1.**
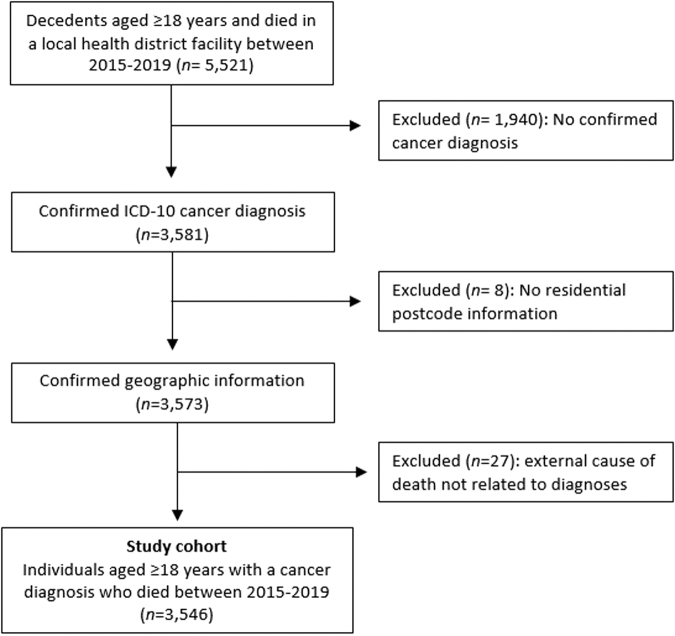
Study cohort.

Data were included for all decedents with a cancer diagnosis excluding non-melanoma skin cancer as defined by diagnostic codes according to the International Statistical Classification of Diseases and Related Health Problems, 10th revision (ICD-10AM)^23^ (Supplementary Table S1), for a 12 month period before death. Data were excluded based on the following criteria: age at death ≤18 years, no cancer diagnosis, reside outside the LHD catchment, or cause of death unrelated to diagnosis (external). A total of 3546 decedents were included in the analysis (Fig. 1).

### Dependent variables (outcomes)

Outcomes were measures of inpatient and outpatient health care services used in the last 12 months of life. Six inpatient services were categorized by intent. Intent was categorized as acute care (curative/life sustaining) or palliative care (PC). Conditional rates of services per 10,000 population were calculated as the number of visits per health care service by statistical area level 2 (SA2)^24^ population extracted from the Australian Bureau of Statistics (ABS) multiplied by 10,000.

Inpatient acute-care services included the following: acute hospital admissions (inpatient episodes recorded as acute), ED visits, ICU admissions, and mechanical ventilation (MV). Other inpatient services included the following: inpatient chemotherapy treatment (ICD-10 Z51.1), inpatient radiotherapy treatment (ICD-10 Z51.0). Inpatient palliative care services included the following: inpatient PC defined as admission into one of the designated PC units OR an inpatient episode recorded as “palliative” care type OR ICD-10-AM Z51.5, inpatient SPC defined as a subset of PC involving hospital admission occurring either under a PC specialist and/or in a SPC ward.

Outpatient services included the following: outpatient chemotherapy (“Cancer—Chemotherapy” NAP service type), outpatient radiotherapy (“Cancer—Radiation Oncology” NAP service type), outpatient cancer care (“Oncology,” “Medical Oncology Consultation” NAP service type), and outpatient SPC (“Palliative,” “Palliative-Care Consultation” NAP service type).

In the context of this study, higher usage rates of acute-care services such as ED visits are often considered indicators of poor-quality EOL care.^25^ In addition, chemotherapy and radiotherapy treatments are often provided with acute or curative/life-sustaining intent or palliative intent. As it was not possible to determine their intent using our data sources, inpatient chemotherapy and radiotherapy services were not classified as acute or palliative, which will require some care when interpreting the results. All outpatient services were categorized by “outpatient” service delivery type.

### Measures of geographic remoteness

Reflecting a large urban footprint, the LHD covers an area >50,000 km^2^. With an average population of 3000 to 25,000 persons per statistical area 2 (SA2) across Australia,^24^ our sample covered 36 SA2s, which had a mean population of 98.5 ± 81.9 and an average spatial area of 3658 km^2^.

Modified Monash Model (MMM) index was used to measure rurality (based on geographic remoteness and population size)^26^ using decedent's residential postcodes mapped to SA2 data^24^ extracted from the ABS. MMM categories included metropolitan areas (MMM1), regional centers (MMM2), large rural towns (MMM3), medium rural towns (MMM4), and small rural towns (MMM5).

Using each decedent's linked SA2 code (according to the postcode of the last known place of residence) and a facility identification code (according to the hospital associated with their inpatient or outpatient record), the shortest travel time to the hospital visited was estimated, using the “Travel-time to hospital in Australia” dataset. This dataset provides estimates of the SA2 population average of shortest car travel times to any hospital in Australia.^27^

### Covariates

Analyses were adjusted for age at death (18–44, 45–54, 55–64, 65–74, 75–84, 85+ years), sex (male, female), marital status (married/not married), preferred language (English/non-English), area-level socioeconomic status,^28^ Charlson Comorbidity Index (CCI),^29^ and cancer type (Supplementary Table S1).

### Statistical analysis

Descriptive statistics were used to summarize covariates and outcomes. Chi-square tests were used to assess differences by rurality in health care utilization rates across service types by care intent. The effects of the rurality and travel-time estimates measures were evaluated with multivariate logistic regression using a log link function and negative binomial and zero-inflated distributions to estimate the adjusted rate ratios (aRR) and their 95% confidence intervals (CIs), for each of the dependent variables in the model. All regression models were adjusted by the covariates. All analyses were performed using SAS 9.4.

### Sensitivity analysis

As advanced cancer patients tend to experience increased rates of health care utilization in their last stages of life,^30^ a sensitivity analysis was conducted to control for potential bias caused by these spikes of health service usage. Excluding the last hospital stay before death from analyses, the effects of rurality and travel time were assessed in a subgroup of decedents who received inpatient PC and SPC services in the time before the last hospital stay before death.

This research received approval from the Health and Medical Ethics Committee of the University of Wollongong (Reference number: 2020/ETH00313).

## Results

The study cohort included 3546 decedents aged ≥18 years. At time of death, the majority of decedents were male (60.1%), aged ≥65 years (77.1%), married (56.9%), and had a CCI of 5+ (78.2%). The number of cases with lung cancer (18.2%) was highest among all cancer diagnoses included in this study. Over 63% of the cohort resided in a metropolitan area and the majority of the population lived within 5–<10 minutes travel time of a public hospital (Table 1).

**Table 1.** Demographic and Clinical Characteristics of Study Cohort (*N* = 3546)

Characteristics	*N*	%
Total	3546	100
Sex		
Female	1416	39.9
Male	2130	60.1
Age (years)		
18–44	68	1.9
45–54	200	5.6
55–64	545	15.4
65–74	953	26.9
75–84	1078	30.4
85+	702	19.8
Marital status		
Married	2018	56.9
Not married	1528	43.1
Preferred language		
English	3272	92.3
Non-English	274	7.7
Cancer type		
Endocrine	19	0.5
>1 cancer type^*^	57	1.7
Gynecological	81	2.3
Brain/CNS	95	2.7
Head & neck	106	3.0
Melanoma	119	3.4
Breast	212	5.9
Prostate	213	6.0
Pancreas	215	6.1
Genitourinary	228	6.4
Colorectal	329	9.3
GI noncolorectal	358	10.1
Hematologic	419	11.8
Other^**^	448	12.6
Lung	647	18.2
CCI		
0–2	189	5.4
3–4	583	16.4
5+	2774	78.2
SEIFA		
Most disadvantaged	943	26.6
More disadvantaged	898	25.3
Average	995	28.1
Less disadvantaged	660	18.6
Least disadvantaged	50	1.4
MM index		
Metropolitan	2239	63.1
Regional centers	149	4.2
Large rural towns	445	12.6
Medium rural towns	566	16.0
Small rural towns	147	4.1
Travel time (minutes)		
0–<5	379	10.6
5–<10	951	26.8
10–<15	608	17.2
15–<30	917	25.9
30+	691	19.5
Acute-care services		
Acute hospitalization	2918	82.3
ED visit	3359	94.7
ICU admission	333	9.4
Mechanical ventilation	110	3.1
Inpatient chemotherapy and radiotherapy		
Inpatient chemotherapy	285	8.1
Inpatient radiotherapy	301	8.5
Palliative care services		
Inpatient palliative care	3515	99.1
Inpatient palliative care before last stay (SA)	2705	76.3
Inpatient specialist palliative care	2025	57.1
Inpatient specialist palliative care before last stay (SA)	284	8.1
Outpatient services		
Outpatient specialist palliative care	1693	47.7
Outpatient chemotherapy	1237	34.8
Outpatient radiotherapy	1011	28.5
Outpatient cancer services	1426	40.2

^*^ “>1 Cancer type” refers to >1 primary cancer site declared in health record.

^**^ “Other” includes all invasive cancer sites not specified above starting with “C” in ICD-10 and excludes nonmelanoma skin cancer.

CNS, central nervous system; CCI, Charlson comorbidity index; SEIFA, socioeconomic index for areas; MM, modified monash; ED, emergency department; ICU, intensive care unit; SA, sensitivity analysis.

### Inpatient services with acute-care intent in the last 12 months of life

In the 12 months preceding death, 82.3% of the study cohort had ≥1 acute hospital admission, 94.7% had ≥1 ED visit, 9.4% had ≥1 ICU admission, and 3.1% had ≥1 hospital stay receiving MV (Table 1). Bivariate analyses found conditional rates of acute hospital admissions and ED visits to vary by rurality (Table 2) and rates of acute hospital admissions to vary by travel time (Table 3).

**Table 2.** Proportion of Decedents With Any Inpatient or Outpatient Health Care Service Use by Service Type and Rurality (Conditional Rates per 10,000 Population)

		Rurality (MM)					
Service type^#^	Total sample (%) (*n* = 3546)	Metropolitan (%) (*n* = 2239)	Regional center (%) (*n* = 149)	Large rural town (%) (*n* = 445)	Medium rural town (%) (*n* = 566)	Small rural town (%) (*n* = 147)	*p*- Value
Acute-care services							
Acute hospital admission	82.3	64.4 (2.0 visits)	3.9 (1.6 visits)	11.7 (1.7 visits)	15.9 (1.8 visits)	4.1 (1.7 visits)	<0.0001
Emergency department visit	94.7	63.0 (3.0 visits)	3.9 (2.5 visits)	12.7 (3.4 visits)	16.3 (4.0 visits)	4.1 (3.3 visits)	0.0006
ICU admission	9.4	61.3 (0.09 visits)	5.1 (0.12 visits)	12.0 (0.10 visits)	15.6 (0.11 visits)	6.0 (0.16 visits)	0.1299
Mechanical ventilation	3.1	62.7 (0.03 visits)	5.5 (0.04 visits)	11.8 (0.03 visits)	14.5 (0.02 visits)	5.5 (0.05 visits)	0.0614
Inpatient chemotherapy and radiotherapy services							
Inpatient chemotherapy	8.1	64.9 (0.11 visits)	6.0 (0.13 visits)	11.9 (0.10 visits)	13.7 (0.08 visits)	3.5 (0.09 visits)	0.9225
Inpatient radiotherapy	8.5	80.1 (0.12 visits)	3.3 (0.07 visits)	5.6 (0.04 visits)	10.3 (0.06 visits)	0.7 (0.01 visits)	<0.0001
Inpatient palliative care services							
Inpatient palliative care	99.1	63.1 (2.4 stays)	4.2 (2.1 stays)	12.5 (2.3 visits)	16.0 (2.3 visits)	4.0 (2.2 visits)	0.7225
Inpatient palliative care before last stay^a^	76.3	64.2 (1.5 stays)	3.9 (1.2 stays)	12.3 (1.4 visits)	15.6 (1.4 visits)	4.0 (1.3 visits)	0.1457
Inpatient SPC	57.1	65.1 (0.67 stays)	4.7 (0.7 visits)	13.3 (0.72 visits)	12.6 (0.5 stays)	4.3 (0.6 visits)	<0.0001
Inpatient SPC before last stay^a^	8.1	60.9 (0.09 stays)	3.2 (0.09 visits)	17.6 (0.14 visits)	15.1 (0.09 visits)	3.2 (0.07 visits)	0.0860
Outpatient services							
Outpatient specialist palliative care	47.7	59.2 (2.2 visits)	4.1 (1.6 visits)	15.0 (3.1 visits)	17.6 (2.9 visits)	4.1 (2.3 visits)	<0.0001
Outpatient chemotherapy	34.8	46.0 (6.0 visits)	3.4 (5.7 visits)	19.3 (6.7 visits)	25.2 (6.5 visits)	6.1 (7.1 visits)	<0.0001
Outpatient radiotherapy	28.5	55.3 (5.8 visits)	4.3 (5.0 visits)	15.8 (7.6 visits)	19.8 (6.7 visits)	4.8 (5.8 visits)	<0.0001
Outpatient cancer care	40.2	52.0 (1.7 visits)	4.0 (1.8 visits)	17.2 (4.3 visits)	21.1 (3.9 visits)	5.7 (3.8 visits)	<0.0001

# Patients can receive ≥1 health service (not mutually exclusive).

Values are percentages of total sample population who had ∼1 health service (conditional rates per 10,000 population).

^a^ Calculated for stays not including the last seven days before death.

**Table 3.** Proportion of Decedents With Any Inpatient or Outpatient Health Care Service Use by Service Type and Travel Time

		Travel time						
Service type^#^	Total sample (%) (*n* = 3546)	Health care facility	0– < 5 min* n *(%)	5– < 10 min* n *(%)	10– < 15 min* n *(%)	15– < 30 min* n *(%)	30+ min* n *(%)	*p*-Value
Acute-care services								
Acute hospital admission	82.3	Nearest acute-care facility	379 (10.8)	951 (26.9)	608 (17.7)	917 (25.5)	691 (19.1)	0.0209
Emergency department visit	94.7	Nearest ED facility	608 (17.2)	1612 (45.8)	555 (15.6)	409 (11.2)	362 (10.2)	0.2016
ICU admission	9.4	Nearest ICU facility	379 (7.8)	951 (26.4)	608 (17.4)	917 (27.3)	691 (21.1)	0.4495
Mechanical ventilation	3.1	Nearest MV facility	379 (11.8)	951 (27.3)	608 (14.5)	917 (28.2)	691 (18.2)	0.9205
Inpatient chemotherapy and radiotherapy services								
Inpatient chemotherapy	8.1	Nearest chemotherapy facility	379 (9.8)	951 (27.0)	608 (14.1)	917 (32.3)	691 (16.8)	0.0908
Inpatient radiotherapy	8.5	Nearest radiotherapy facility	379 (14.6)	951 (22.6)	608 (20.6)	917 (29.6)	691 (12.6)	0.0008
Inpatient palliative care services								
Inpatient palliative care	99.1	Nearest palliative care facility	781 (22.0)	784 (22.0)	879 (24.9)	506 (14.3)	596 (16.8)	0.3041
Inpatient palliative care before last stay^a^	76.3	Nearest palliative care facility	781 (22.1)	784 (21.6)	879 (25.7)	506 (14.4)	596 (16.2)	0.1200
Inpatient SPC	57.1	Nearest SPC facility	781 (23.5)	423 (12.1)	879 (25.6)	756 (22.1)	707 (16.7)	<0.0001
Inpatient SPC before last stay^a^	8.1	Nearest SPC facility	781 (19.0)	423 (9.2)	879 (27.1)	756 (27.1)	707 (17.6)	0.0470
Outpatient services								
Outpatient specialist palliative care	47.7	Nearest SPC facility	781 (19.4)	784 (23.9)	879 (23.5)	506 (14.8)	596 (18.4)	0.0002
Outpatient chemotherapy	34.9	Nearest chemotherapy facility	379 (6.1)	951 (27.1)	608 (14.0)	917 (24.7)	691 (28.1)	<0.0001
Outpatient radiotherapy	28.5	Nearest radiotherapy facility	379 (8.6)	951 (26.8)	608 (16.1)	917 (25.4)	691 (23.1)	0.0031
Outpatient cancer care	40.2	Nearest cancer care facility	379 (7.5)	951 (25.4)	608 (16.1)	917 (26.5)	691 (24.5)	<0.0001

# Patients can receive ≥1 health service (not mutually exclusive).

Values are percentages of total sample population who had ∼1 health service.

^a^ Calculated for stays not including the last seven days before death.

In multivariate analyses, rurality was associated with higher rates of acute-care services with decedents living in small rural towns having 1.3 times the adjusted rates of having ≥1 ED visit (aRR 1.294, 95% CI: 1.065–1.571) and 2.0 times the adjusted rates of receiving MV more than once (aRR 2.020, 95% CI: 1.211–3.368) in the last 12 months of life compared with urban counterparts. Decedents traveling 30+ minutes to their nearest ED department were less likely to have ≥1 ED visit in their last year of life compared with those living closest (0–<5 minutes) (aRR 0.670, 95% CI: 0.583–0.769) (*p* < 0.0001), a conflicting result to the MM index finding (Table 4).

**Table 4.** Adjusted Rate Ratios for Association of Geographic Factors Influencing Inpatient Services in the Last 12 Months of Life

	Adjusted rate ratio (95% confidence interval)					
	≥1 Acute hospitalization	≥1 ED visit	≥1 ICU admission	≥1 Mechanical ventilation	≥1 Chemotherapy treatment^^^	≥1 Radiotherapy treatment*^a^*
Characteristics						
MM index						
Metropolitan	1.0	1.0	1.0	1.0	1.0	1.0
Regional centers	0.858 (0.726–1.014)	0.876 (0.745–1.032)	1.208 (0.761–1.918)	**2.198 (1.299**–**3.719)**	1.208 (0.824–1.770)	**0.534 (0.303**–**0.942)**
Large rural towns	**0.825 (0.756**–**0.900)**^b^	1.067 (0.973–1.171)	**1.317 (1.026**–**1.692)**	0.951 (0.697–1.298)	0.915 (0.730–1.146)	**0.320 (0.231**–**0.444)**^b^
Medium rural towns	0.947 (0.801–1.119)	**1.660 (1.478**–**1.865)**	1.174 (0.734–1.880)	1.695 (0.985–2.917)	0.890 (0.568–1.394)	**0.367 (0.193**–**0.699)**
Small rural towns	0.888 (0.758–1.040)	**1.294 (1.065**–**1.571)**	1.486 (0.965–2.286)	**2.020 (1.211**–**3.368)**	0.669 (0.435–1.029)	**0.071 (0.028**–**0.180)**^b^
Travel time (mins)^c^						
0–<5	1.0	1.0	1.0	1.0	1.0	1.0
5–<10	1.025 (0.932–1.127)	1.066 (0.986–1.153)	1.155 (0.849–1.572)	0.831 (0.590–1.169)	0.969 (0.740–1.268)	**0.737 (0.556**–**0.975)**
10–<15	1.048 (0.957–1.147)	0.985 (0.899–1.079)	1.282 (0.953–1.724)	0.708 (0.501–1.001)	0.892 (0.684–1.164)	0.914 (0.710–1.176)
15–<30	0.973 (0.890–1.064)	0.930 (0.805–1.075)	1.212 (0.910–1.616)	0.834 (0.605–1.151)	1.186 (0.931–1.510)	1.009 (0.793–1.285)
30+	0.967 (0.818–1.143)	**0.670 (0.583**–**0.769)^b^**	1.128 (0.703–1.811)	0.580 (0.337–1.000)	0.853 (0.540–1.348)	0.956 (0.516–1.773)

^a^ Inpatient chemotherapy and inpatient radiotherapy services have been included in this table for convenience, and not necessarily categorized as services with acute-care intent.

Where *p* < 0.05, figures are in bold, ^b^indicates statistical significance (*p* < 0.0001).

Adjusted for sex, age at death, marital status, preferred language, cancer type, comorbidity index, socioeconomic status, rate ratio from negative binomial regression for health care utilization with count data.

^c^ Nearest facility with health service (e.g., emergency department, intensive care unit, specialist palliative care unit).

### Inpatient chemotherapy and radiotherapy services in the last 12 months of life

In the last 12 months of life, 8.1% of the cohort had ≥1 hospital stay receiving chemotherapy treatment and 8.5% had ≥1 hospital stay receiving radiotherapy treatment (Table 1). Correlated to rurality and travel time, bivariate analyses found rates of inpatient radiotherapy lowest among those living in small rural towns (Table 2) and those with a travel time of >30 minutes (Table 3).

In multivariate analyses, rurality was associated with lower rates of inpatient radiotherapy with decedents living in small rural towns having the lowest rates compared with metropolitan residents (aRR 0.071, 95% CI: 0.028–0.180). Similar to the effects found with rurality, longer travel time to inpatient radiotherapy hospital-based facilities was associated with lower rates of inpatient radiation therapy treatment (aRR 0.737, 95% CI: 0.556–0.975), with similar patterns found for inpatient chemotherapy, although not significant (Table 4).

### Inpatient services with palliative-care intent in the last 12 months of life

Across all inpatient hospital stays in the last 12 months of life, 99.1% of the cohort had ≥1 stay receiving PC and 57.1% had ≥1 stay receiving SPC. When excluding the last hospital stay before death as part of the sensitivity analysis, the rates of inpatient PC (99.1% to 76.3%) and inpatient SPC treatment (57.1% to 8.1%) significantly reduced (Table 1). Bivariate analyses found only conditional rates of inpatient SPC to be correlated to rurality with the lowest rates among decedents living in small rural towns (0.6 visits per 10,000 population) (Table 2). Travel time was also correlated to inpatient SPC with lowest rates found among decedents with a 5-<10 minutes travel time to the nearest SPC facility (Table 3).

In multivariate analysis, rurality was associated with lower rates of inpatient PC with decedents living in regional centers having lower rates of PC when including (aRR 0.851, 95% CI: 0.746–0.971) and excluding (aRR 0.754, 95% CI: 0.600–0.947) the last hospital stay (*p* < 0.05) compared with metropolitan residents. The measurement of rurality according to MM index was unable to predict rates of inpatient SPC when including and excluding the last hospital admission.

Shorter travel times to the nearest SPC facility of 10–<15 minutes (aRR 1.276, 95% CI: 1.001–1.626) and 15–<30 minutes (aRR 1.477, 95% CI: 1.098–1.986) were correlated to higher rates of SPC in the last 12 months of life, when excluding the last hospital stay (*p* < 0.05) (Table 5). Rates of inpatient PC and SPC when including the last hospital admission in the analysis did not vary by travel time.

**Table 5.** Adjusted Rate Ratios for Association of Geographic Factors Influencing Inpatient Services With Palliative-Care Intent in the Last 12 Months of Life

	Adjusted rate ratio (95% confidence interval)			
			Sensitivity analysis	
	Inpatient PC (hospital admission coded as palliative care)	Inpatient SPC (hospital admission occurring either under a PC specialist &/or in a SPC ward)	Inpatient PC (received before last stay)	Inpatient SPC (received before last stay)
Characteristics				
MM index				
Metropolitan	1.0	1.0	1.0	1.0
Regional centers	**0.851 (0.746**–**0.971)**	1.114 (0.866–1.433)	**0.754 (0.600**–**0.947)**	0.853 (0.506–1.437)
Large rural towns	0.959 (0.896–1.026)	1.108 (0.950–1.292)	0.940 (0.837–1.056)	1.157 (0.859–1.558)
Medium rural towns	1.008 (0.868–1.171)	0.924 (0.616–1.386)	0.984 (0.760–1.274)	1.091 (0.535–2.224)
Small rural towns	0.892 (0.780–1.021)	1.098 (0.702–1.717)	0.825 (0.655–1.038)	0.759 (0.326–1.765)
Travel time (mins)^a^	PC facility	(SPC facility)	PC facility	(SPC facility)
0–<5	1.0	1.0	1.0	1.0
5–<10	1.000 (0.933–1.071)	0.875 (0.752–1.018)	0.986 (0.877–1.110)	0.742 (0.522–1.054)
10–<15	1.038 (0.982–1.098)	0.996 (0.891–1.114)	1.056 (0.961–1.161)	**1.276 (1.001**–**1.626)**
15–<30	1.045 (0.961–1.135)	0.957 (0.823–1.112)	1.105 (0.962–1.270)	**1.477 (1.098**–**1.986)**
30+	0.965 (0.828–1.126)	0.723 (0.476–1.098)	0.949 (0.728–1.237)	0.761 (0.357–1.623)

Where *p* < 0.05, figures are in bold.

Adjusted for sex, age at death, marital status, preferred language, cancer type, comorbidity index, socioeconomic status, rate ratio from negative binomial regression for health care utilization with count data.

^a^ Nearest facility with health service (e.g., emergency department, intensive care unit, specialist palliative care facility/ward).

PC, palliative care; SPC, specialist palliative care.

### Outpatient services in the last 12 months of life

In the last 12 months of life, 47.7% of decedents used outpatient SPC services, 34.8% used outpatient chemotherapy services, 28.5% used outpatient radiotherapy services, and 40.2% used outpatient cancer services (Table 1). Bivariate comparisons showed rurality and travel time to be correlated with all outpatient services (*p* < 0.0001) (Tables 2 and 3). Multivariate analyses found rurality to be strongly associated with lower rates of outpatient chemotherapy; decedents living in large rural towns (aRR 0.474, 95% CI: 0.371–0.606), medium rural towns (aRR 0.324; 95% CI: 0.203–0.671), and small rural towns (aRR 0.432, 95% CI: 0.278–0.671) were significantly less likely to use outpatient chemotherapy treatment compared with metropolitan residents.

Decedents living in regional centers also had lower rates of outpatient radiotherapy service usage (aRR 0.510, 95% CI: 0.287–0.907), whereas decedents living in large rural towns were 1.8 times more likely to use outpatient cancer care services (aRR 1.804, 95% CI: 1.457–2.235) (*p* < 0.0001) compared with metropolitan dwellers (Table 6). Travel time to outpatient services including SPC, chemotherapy, radiotherapy, and cancer care showed no impact on usage rates.

**Table 6.** Adjusted Rate Ratios of Geographic Factors Influencing Outpatient Services in the Last 12 Months of Life

	Adjusted rate ratio (95% confidence interval)			
	Outpatient SPC	Outpatient chemotherapy treatment	Outpatient radiotherapy treatment	Outpatient cancer care
Characteristics				
MM index				
Metropolitan	1.0	1.0	1.0	1.0
Regional centers	0.743 (0.493–1.118)	0.716 (0.441–1.162)	**0.510 (0.287**–**0.907)**	0.892 (0.586–1.358)
Large rural towns	1.133 (0.939–1.367)	**0.474 (0.371**–**0.606)**^a^	0.842 (0.604–1.172)	**1.804 (1.457**–**2.235)**^a^
Medium rural towns	1.305 (0.826–2.063)	**0.324 (0.203**–**0.517)**^a^	0.539 (0.291–1.001)	1.246 (0.851–1.826)
Small rural towns	1.219 (0.817–1.819)	**0.432 (0.278**–**0.671)**	0.624 (0.348–1.116)	1.320 (0.910–1.916)
Travel time (mins)^b^				
0–<5	1.0	1.0	1.0	1.0
5–<10	0.899 (0.730–1.107)	1.108 (0.772–1.591)	1.048 (0.694–1.583)	0.946 (0.697–1.283)
10–<15	0.841 (0.700–1.011)	0.938 (0.661–1.329)	0.857 (0.589–1.245)	0.958 (0.725–1.264)
15–<30	0.863 (0.670–1.113)	0.988 (0.700–1.393)	1.061 (0.742–1.517)	1.000 (0.763–1.311)
30+	0.872 (0.544–1.396)	1.393 (0.838–2.316)	1.514 (0.809–2.833)	1.202 (0.792–1.825)

Where *p* < 0.05, figures are in bold, ^a^indicates statistical significance (*p* < 0.0001),

Adjusted for sex, age at death, marital status, preferred language, cancer type, comorbidity index, socioeconomic status, rate ratio from zero-inflated negative binomial regression (count model) for health care utilization with count data.

^b^ Nearest facility with health service (e.g., outpatient chemotherapy facility, outpatient oncology unit).

SPC, specialist palliative care.

### Covariates associated with outcomes

Older age (≥75 years) was associated with lower rates of inpatient and outpatient services except those aged ≥45 years had increased rates of outpatient radiotherapy (*p* < 0.05). Female decedents had lower rates of acute-care service usage including ED visits (aRR 0.933, 95% CI: 0.882–0.987) yet higher rates of inpatient SPC when including (aRR 1.140, 95% CI: 1.051–1.237) and excluding (aRR 1.217, 95% CI: 1.024–1.447) the last hospital stay before death (*p* < 0.05). Being unmarried reduced the odds of inpatient PC, inpatient SPC when received before the last stay, outpatient SPC, and outpatient chemotherapy services.

Hematologic cancer was associated with higher rates of acute hospital admissions (aRR 1.185, 95% CI: 1.054–1.331), ICU admissions (aRR 5.886, 95% CI: 3.493–9.920), inpatient chemotherapy (aRR 9.059, 95% CI: 5.418–15.145), MV (aRR 10.684, 95% CI: 4.354–26.220), and outpatient chemotherapy services (aRR 3.002, 95% CI: 2.040–4.417). Brain/CNS and head & neck cancer types were also associated with increased rates of ICU admissions, MV, and outpatient radiotherapy (*p* < 0.001), and lung cancer diagnosis was positively correlated to rates of inpatient chemotherapy, MV, and outpatient radiotherapy (*p* < 0.05). CCI score was positively correlated to rates of acute hospital admissions, inpatient chemotherapy, inpatient radiotherapy, PC and SPC across all hospital stays, but inversely correlated to ED visits in the last year of life (Supplementary Tables S2–S7).

## Discussion

This study assessed the effects of geographic remoteness using two measures (rurality and travel time) on rates of inpatient and outpatient EOL care service utilization among advanced cancer patients in an Australian LHD. Significant regional variation was observed for indicators of inpatient acute care with patients living in least populated, rural regions more likely to have an ED visit, ICU admission, and MV intervention in their last 12 months of life compared with those in urban areas.

Travel time (10–<30 minutes) to the nearest hospital was a significant predictor of increased rates of inpatient SPC. Being male, not married, cancer type of hematologic, brain/CNS and head & neck cancer types, and presence of multimorbidities were related to increased use of inpatient acute-care services. Being female, younger age, not married, and presence of multimorbidities were related to higher rates of inpatient PC service usage. Being older age (≥75 years) and unmarried significantly increased the use of outpatient services.

### Inpatient services with acute-care intent

Rurality was associated with lower rates of acute hospitalizations, but higher rates of ED visits, ICU admissions, and MV. Conversely, a longer travel time was associated with lower rates of ED visits. Previous studies showed similarly mixed findings, with a North American study reporting less hospitalizations with increasing rurality,^31^ while other studies from Germany and Canada reported higher rates of hospitalizations.^13,32,33^ International studies examining the association between rurality and ED visits also showed conflicting findings with studies showing both higher^32^ and lower^31^ rates. Mixed findings were also seen in previous studies examining the association between rurality and ICU admissions and MV.^30,32,34^

The variation by rurality and travel time in rates of ED visits uncovered in our study is interesting, and may be related to regional differences across the LHD in availability of nonhospital-based EOL health care resources such as after-hours GP,^35^ or be explained by rural residents being more prepared to travel for urgent medical care at times of need. Further investigation into area-level primary health care supply would help better understand this association. The heterogeneity between our findings and those of international studies also highlights the methodological challenges^36^ involved when comparing studies that use different course geographic access measures.

Studies solely measuring rurality may underestimate geographic barriers to accessing health care, especially if there is variability in area size, population density, and distance to urban resources. Differences in the way U.S., Canadian, and Australian health care systems are organized, including insurance coverage policies,^1^ may also be influencing the types of services used and patient preferences.^2^

Low rates of hospitalization by rural decedents in our study may reflect important within-country differences, including the impact of government initiatives in partnership with LHDs that were implemented between 2012 and 2016 with the aim of expanding outpatient and at-home PC services in rural areas,^6^ keeping more rural patients out of hospital.^34^ It is plausible for the impact of such initiatives to not be captured in our travel-time estimates. The vastness of the LHD, road closures, and public transport networks means that some people living in urban or regional areas may experience extended travel times, which are not dissimilar to, or even longer than for many people living in rural areas.

### Inpatient chemotherapy and radiotherapy services

Increasing rurality and travel times were associated with lower rates of inpatient radiotherapy treatment with decedents living in small rural towns significantly less likely than metropolitan residents to receive radiotherapy treatment during an inpatient hospital admission. Similar patterns, although not significant, were observed for rates of inpatient chemotherapy.

Although these numbers only reflect the last 12 months of the patient's life, the undersupply of radiotherapy and chemotherapy facilities across the LHD may be contributing to an underutilization of these health care services with many rural patients often declining treatment if they are unable to travel significant distances for sometimes weeks' worth of treatment.^37^ With similar reduced rates of survival found among colorectal patients living >100 km away from a radiotherapy center in Queensland,^38^ establishing satellite chemotherapy and radiotherapy treatment centers across rural and regional regions presents as a tangible solution to improve access for rural patients.

### Inpatient services with PC intent

Encouragingly, almost all patients in the cohort received PC treatment at least once during a hospital admission coded “palliative” in the last 12 months of life. In comparison, only 57% of the cohort received SPC; that is, a hospital admission occurring either under a PC specialist and/or in a SPC ward. Although these SPC results reflect similar usage rates found among advanced cancer patients in Western Australia,^39^ one-third of our cohort did not receive SPC. While some may not have needed SPC^39^ or received it at home, referral patterns, workforce shortages, and limited supply of only two designated SPC wards in the region may be creating an “access abyss,”^40^ which requires additional resources to meet the unmet need. Living in a regional center was associated with lower rates of inpatient PC, with no effect on inpatient SPC.

As rates of inpatient PC or SPC did not vary by travel-time estimates despite there being high usage rates of PC, a sensitivity analysis was conducted to control for the fact that the last hospital admission for each patient (i.e., the admission when the patient died) was usually coded as inpatient PC or SPC. When conducted, the effects of rurality remained consistent; however, decedents with a long travel time (30 minutes+) appear to have lower rates of inpatient SPC (but not all inpatient PC) than those living closer (10–<30 minutes) to hospital. This finding suggests travel time to a SPC unit acts as a significant barrier to patients accessing SPC, and that rurality and travel times to the nearest hospital act as a significant barrier to inpatient PC.

Previous studies conducted in Australia and the United States showed similar findings in relation to SPC^39,41^ but did not examine nonspecialist inpatient PC. Although the cost and governance of hospice and PC services differ between Australia and the United States, the strong distance decay association linked to inpatient SPC found in both countries highlights the significant impact travel burden has on rural cancer patients living in countries with similar geographic spread.

Although more research that encompasses both public- and private-funded PC is needed to gather a complete picture, the observed disparity suggests that policies aimed at reducing travel times to inpatient SPC facilities are needed in rural and remote communities to increase the provision of PC, thereby reducing the “rural tax” associated with many rural cancer patients needing to leave their communities to seek inpatient PC care in city centers^42^; An important factor to consider, especially when developing policies to address inequalities that are not traditionally underpinned by health status or financial barriers.

Other independent risk factors associated with not receiving PC included being older (>85 years), male, and not being married. Our findings need to be considered with caution as we were unable to capture those who may have died outside of hospital or received PC at home.^39^ The differences in uptake of PC according to gender and marital status is not surprising, potentially reflecting male stoicism at EOL^43^ and the dependency that people without spouses may have on family or friends to provide travel, support, and visitation during treatments.^39^ These are concerning findings that call for increased PC support for people living in rural and regional communities, especially if they are older, male, and single.

### Outpatient services

Rurality was a significant predictor of low rates of outpatient chemotherapy and radiotherapy treatment. Decedents living in regional centers and rural towns received chemotherapy and radiotherapy treatment as an outpatient at a significantly lower rate than those who lived in the metropolitan areas of the LHD. Rurality was also associated with higher rates of outpatient cancer care services, with decedents from large rural towns having 1.8 times the likelihood of accessing outpatient cancer services compared with urban residents.

In contrast to rurality, travel-time estimates were not associated with any differences in the utilization rates of any of the outpatient services in the last year of life. Considering similar results have also been reported in a study of lung cancer patients in the United States,^13^ the use of outpatient services among advanced cancer patients may depend more on patient-level factors than on geographic or health care system factors, especially considering the health system differences between Australia and the United States. Although indicative of just the last 12 months of life, the low usage rates of outpatient chemotherapy and radiotherapy in this study may be explained by the concept of therapeutic refusal.

Considering the demographics (older age, unmarried^44^) and clinical characteristics (cancer type^45^) of the study cohort, many patients may have declined treatment due to the desire not to undergo treatments without a curative proposal^46^ or the fear of side effects.^47^ From a health system perspective, the higher rates of outpatient SPC and outpatient cancer services used among rural patients may reflect the quality of outpatient cancer services being delivered to patients at home across this LHD, helping to reduce the barriers of geographic access.

### Strengths and limitations

As prior research has shown geographic access to be a strong determinant of health care use and health outcomes,^48–50^ the number of studies exploring this relationship in the context of palliative and EOL care for advanced cancer patients is growing. However, unlike prior studies relying on rurality or distance as sole measure of geographic access, this study contributes to an emerging body of geography-based research on EOL cancer care, which combine urban/rural characteristics of patient residence with distance/travel time to health care facilities to measure geographic access.^19,51–53^

To our knowledge, this is the first application of such analytical methods in a study exploring EOL inpatient and outpatient cancer care service utilization for a LHD in Australia. Along with controlling for a large number of covariates and the use of individual-level data to minimize the spatial analysis issue of modifiable areal unit,^54^ our study showed the travel-time estimation method to be relatively straightforward to apply and represents a useful analytical tool when used in conjunction with measures of rurality to assess the effects of geographic access on health care use.

Nevertheless, measures of both rurality and travel times sometimes produced conflicting and confusing results, and an extension of the categories to better assess travel times of >30 mins in rural areas would help reduce future measurement bias. Prior studies have also reported area-level workforce supply^51,55^ and patient preferences^56,57^ as important factors in determining the care received at EOL. We did not have information on the decedents' advance care plans or preferences, and whether these influenced the type and frequency of services used.

While this study was designed to assess the effects of geographic remoteness on the provision of EOL care among advanced cancer patients, assessing the quality and appropriateness of this medical care, or lack thereof, is also of significance. Future studies that incorporate data sources that capture the intent of health care services such as chemotherapy and radiotherapy may assist researchers to further analyze the quality of care received at EOL.

## Conclusions

Using data from a geographically diverse Australian LHD, this study examined the effects of geographic remoteness using measures of rurality and travel time on EOL care service utilization among advanced cancer patients. Reporting on a series of inpatient and outpatient services used in the last year of life, our findings suggest that rurality and travel-time estimates can be useful tools to estimate geographic variation in EOL cancer care provision, with significant gaps uncovered in inpatient PC and outpatient service utilization in rural areas.

Rurality was consistently associated with increased use of inpatient services with acute-care intent, and shorter travel time (10–<30 minutes) was a significant predictor of increased use of inpatient PC. This finding has significant implications for policymakers, and suggests initiatives that redistribute EOL resources in rural areas to reduce travel times to health care facilities could help to reduce geographic disparities and optimize access to EOL care services.

## Supplementary Material

Supplemental data

Supplemental data

Supplemental data

Supplemental data

Supplemental data

Supplemental data

Supplemental data

## Accessibility of Data

The data that support the findings of this study are available on request from the corresponding author, J.C., and from the Centre for Health Research Illawarra Shoalhaven Population (CHRISP). Restrictions apply to the availability of these data, which were used under license for this study.
