# Supplementary material for: Does Geography Play a Role in the Receipt of End-of-Life Care for Advanced Cancer Patients? Evidence from an Australian Local Health District Population-Based Study
Source: J Palliat Med. 2023 Nov 8;26(11):1453–65. doi: 10.1089/jpm.2022.0555 (PMC10658736; doi:10.1089/jpm.2022.0555)
Supplement: Supplemental data [file Supp_TableS1.docx]

**Table S1.** Cancer type ICD-10 diagnostic codes

| **Cancer Type** | **ICD-10 Diagnostic Codes** |
| --- | --- |
| Brain/CNS | C69-72 |
| Breast (female) | C50 |
| Breast (in-situ) | D05 |
| Colorectal | C18-C20, C26 |
| Endocrine | C73-75 |
| GI non-colorectal | C15, 16, 17, 21, 22 |
| Genitourinary | C51-68 |
| Gynaecological | C53, C55, C56 |
| Head & Neck | C00-14, C30-32 |
| Hematologic | C81-86, 88, 90-96 |
| Lung | C33-C34 |
| Melanoma | C43 |
| Other | C23-24, C37-41, 45-49, C76-80, 97, D37-49 |
| Pancreas | C25 |
| Prostate | C61 |
