# Supplementary material for: Does Geography Play a Role in the Receipt of End-of-Life Care for Advanced Cancer Patients? Evidence from an Australian Local Health District Population-Based Study
Source: J Palliat Med. 2023 Nov 8;26(11):1453–65. doi: 10.1089/jpm.2022.0555 (PMC10658736; doi:10.1089/jpm.2022.0555)
Supplement: Supplemental data [file Supp_TableS2.docx]

**Table S2.** Adjusted rate ratios of acute inpatient End-of-life care services and associated geographic and socio-demographic factors

| Characteristic | >1 Acute hospitalisation in the last 12 months |  | >1 ED visit in the last 12 months |  | >1 ICU admission in the last 12 months |  |
| --- | --- | --- | --- | --- | --- | --- |
|  | aRR (95% CI) | *P* value | aRR (95% CI) | *P* value | aRR (95% CI) | *P* value |
| Sex |  |  |  |  |  |  |
| Male | 1.0 |  | 1.0 |  | 1.0 |  |
| Female | 1.006 (0.956, 1.059) | 0.8059 | 0.933 (0.882, 0.987) | **0.0166** | 0.878 (0.753, 1.024) | 0.0819 |
| Age (years) |  |  |  |  |  |  |
| 18-44 | 1.0 |  | 1.0 |  | 1.0 |  |
| 45-54 | 0.978 (0.825, 1.160) | 0.8041 | 1.026 (0.836, 1.259) | 0.8034 | 0.982 (0.624, 1.545) | 0.9364 |
| 55-64 | 0.837 (0.715, 0.981) | **0.0287** | 0.898 (0.743, 1.085) | 0.2676 | 0.705 (0.460, 1.078) | 0.0921 |
| 65-74 | 0.798 (0.683, 0.931) | **0.0043** | 0.879 (0.730, 1.058) | 0.1753 | 0.851 (0.563, 1.287) | 0.4056 |
| 75-84 | 0.708 (0.606, 0.827) | **<.0001** | 0.829 (0.689, 0.998) | **0.0487** | 0.398 (0.261, 0.609) | **<.0001** |
| 85+ | 0.589 (0.502, 0.692) | **<.0001** | 0.762 (0.630, 0.920) | **0.0048** | 0.149 (0.092, 0.242) | **<.0001** |
| Marital Status |  |  |  |  |  |  |
| Married | 1.0 |  | 1.0 |  | 1.0 |  |
| Not married | 0.955 (0.911, 1.002) | 0.0628 | 0.997 (0.947, 1.05) | 0.9339 | 0.995 (0.862, 1.149) | 0.9492 |
| Preferred Language |  |  |  |  |  |  |
| English | 1.0 |  | 1.0 |  | 1.0 |  |
| Non-English | 1.089 (0.998, 1.188) | 0.0536 | 1.068 (0.969, 1.177) | 0.1793 | 1.205 (0.899, 1.617) | 0.2099 |
| Cancer Type |  |  |  |  |  |  |
| >1 cancer type* | 1.045 (0.856, 1.275) | 0.6643 | 0.983 (0.789, 1.223) | 0.8782 | 3.179 (1.546, 6.537) | **0.0017** |
| Brain/CNS | 0.760 (0.633, 0.911) | **0.0031** | 0.734 (0.606, 0.891) | **0.0017** | 3.174 (1.686, 5.973) | **0.0003** |
| Breast (female) | 0.818 (0.699, 0.957) | **0.0126** | 0.712 (0.601, 0.842) | **<.0001** | 1.859 (0.972, 3.554) | 0.0605 |
| Breast (in-situ) | 0.850 (0.681, 1.060) | 0.1507 | 0.748 (0.586, 0.955) | **0.0199** | 2.056 (0.914, 4.622) | 0.0811 |
| Colorectal | 0.896 (0.792, 1.014) | 0.0837 | 0.791 (0.694, 0.901) | **0.0004** | 2.902 (1.680, 5.015) | **0.0001** |
| Endocrine | 0.847 (0.605, 1.187) | 0.3372 | 0.858 (0.601, 1.224) | 0.4001 | 4.375 (1.741, 10.996) | **0.0017** |
| GI non-colorectal | 0.961 (0.852, 1.083) | 0.5152 | 0.860 (0.757, 0.977) | **0.0206** | 3.216 (1.886, 5.483) | **<.0001** |
| Genitourinary | 1.009 (0.884, 1.152) | 0.8891 | 1.020 (0.889, 1.171) | 0.7707 | 2.230 (1.228, 4.046) | **0.0083** |
| Gynaecological | 0.811 (0.668, 0.984) | **0.0345** | 0.848 (0.695, 1.035) | 0.1053 | 2.693 (1.334, 5.436) | **0.0057** |
| Head & Neck | 0.851 (0.717, 1.009) | 0.0639 | 0.679 (0.564, 0.817) | **<.0001** | 6.453 (3.637, 11.45) | **<.0001** |
| Hematologic | 1.185 (1.054, 1.331) | **0.0043** | 1.005 (0.886, 1.139) | 0.9355 | 5.886 (3.493, 9.920) | **<.0001** |
| Lung | 0.974 (0.872, 1.088) | 0.6477 | 0.882 (0.785, 0.992) | **0.0362** | 1.548 (0.904, 2.651) | 0.1108 |
| Melanoma | 0.835 (0.709, 0.983) | **0.0307** | 0.792 (0.667, 0.939) | **0.0074** | 4.619 (2.584, 8.257) | **<.0001** |
| Other** | 0.921 (0.819, 1.036) | 0.1733 | 0.870 (0.769, 0.983) | **0.0263** | 3.435 (2.022, 5.836) | **<.0001** |
| Pancreas | 0.924 (0.807, 1.057) | 0.2507 | 0.809 (0.699, 0.935) | **0.0043** | 3.089 (1.760, 5.422) | **<.0001** |
| Prostate | 1.0 |  | 1.0 |  | 1.0 |  |
| CCI |  |  |  |  |  |  |
| 0-2 | 1.0 |  | 1.0 |  | 1.0 |  |
| 3-4 | 1.101 (0.967, 1.254) | 0.1425 | 0.767 (0.675, 0.872) | **<.0001** | 0.720 (0.505, 1.025) | 0.0687 |
| 5+ | 1.161 (1.030, 1.308) | **0.0142** | 0.840 (0.749, 0.942) | **0.0031** | 0.717 (0.519, 0.991) | **0.0443** |
| SEIFA |  |  |  |  |  |  |
| Most Disadvantaged | 1.0 |  | 1.0 |  | 1.0 |  |
| More disadvantaged | 1.003 (0.908, 1.108) | 0.9432 | 0.862 (0.775, 0.959) | **0.0063** | 1.197 (0.884, 1.620) | 0.2438 |
| Average | 1.028 (0.960, 1.102) | 0.4213 | 0.892 (0.827, 0.961) | **0.0029** | 1.242 (1.000, 1.543) | **0.0499** |
| Less disadvantaged | 1.012 (0.932, 1.100) | 0.7601 | 0.920 (0.842, 1.005) | 0.0647 | 1.081 (0.835, 1.400) | 0.5527 |
| Least disadvantaged | 1.186 (0.975, 1.441) | 0.0863 | 1.017 (0.784, 1.319) | 0.8978 | 1.493 (0.837, 2.664) | 0.1740 |
| MMM |  |  |  |  |  |  |
| Metropolitan | 1.0 |  | 1.0 |  | 1.0 |  |
| Regional Centres | 0.858 (0.726, 1.014) | 0.0740 | 0.876 (0.745, 1.032) | 0.1143 | 1.208 (0.761, 1.918) | 0.4217 |
| Large rural towns | 0.825 (0.756, 0.900) | **<.0001** | 1.067 (0.973, 1.171) | 0.1622 | 1.317 (1.026, 1.692) | **0.0306** |
| Medium rural towns | 0.947 (0.801, 1.119) | 0.5269 | 1.660 (1.478, 1.865) | **<.0001** | 1.174 (0.734, 1.880) | 0.5016 |
| Small rural towns | 0.888 (0.758, 1.040) | 0.1416 | 1.294 (1.065, 1.571) | **0.0093** | 1.486 (0.965, 2.286) | 0.0717 |
| Travel Time (mins) *** | *Acute Care facility* |  | *ED facility* |  | *ICU facility* |  |
| 0-<5 | 1.0 |  | 1.0 |  | 1.0 |  |
| 5-<10 | 1.025 (0.932, 1.127) | 0.6071 | 1.066 (0.986, 1.153) | 0.1047 | 1.155 (0.849, 1.572) | 0.3564 |
| 10-<15 | 1.048 (0.957, 1.147) | 0.3088 | 0.985 (0.899, 1.079) | 0.7486 | 1.282 (0.953, 1.724) | 0.1003 |
| 15-<30 | 0.973 (0.890, 1.064) | 0.5549 | 0.930 (0.805, 1.075) | 0.3301 | 1.212 (0.910, 1.616) | 0.1880 |
| 30+ | 0.967 (0.818, 1.143) | 0.6986 | 0.670 (0.583, 0.769) | **<.0001** | 1.128 (0.703, 1.811) | 0.6159 |

Rate ratio from Negative Binomial regression for end-of-life care healthcare utilisation with count data

*’>1 Cancer type’ refers to more than 1 primary cancer site declared

**’Other’ includes all invasive cancer sites not specified above starting with ‘C’ in ICD-10 and exclude non-melanoma skin cancer

***nearest facility with health service (e.g., Emergency Department, Intensive Care Unit, Specialist Palliative Care ward)

aRR= adjusted rate ratio, OR=odds ratio, CI=confidence interval, MV=mechanical ventilation
