# Supplementary material for: Does Geography Play a Role in the Receipt of End-of-Life Care for Advanced Cancer Patients? Evidence from an Australian Local Health District Population-Based Study
Source: J Palliat Med. 2023 Nov 8;26(11):1453–65. doi: 10.1089/jpm.2022.0555 (PMC10658736; doi:10.1089/jpm.2022.0555)
Supplement: Supplemental data [file Supp_TableS7.docx]

**Table S7.** Adjusted rate ratios of outpatient chemotherapy services and associated geographic and sociodemographic factors

| Characteristic | Receipt of Outpatient Radiotherapy treatment | | Receipt of outpatient cancer care services | |
| --- | --- | --- | --- | --- |
|  | **Zero-inflation model** | | **Zero-inflation model** | |
|  | aRR (95% CI) | *P* value | aRR (95% CI) | *P* value |
| Intercept | 2.344 |  | 1.195 |  |
| Sex |  |  |  |  |
| Male | 1.0 |  | 1.0 |  |
| Female | 1.258 (1.031, 1.534) | **0.0232** | 0.781 (0.613, 0.994) | **0.0454** |
| Age (years) |  |  |  |  |
| 18-44 | 1.0 |  | 1.0 |  |
| 45-54 | 0.974 (0.484, 1.958) | 0.9424 | 1.355 (0.506, 3.626) | 0.5441 |
| 55-64 | 1.300 (0.686, 2.465) | 0.4201 | 2.356 (0.963, 5.764) | 0.0604 |
| 66-74 | 1.388 (0.741, 2.600) | 0.3050 | 2.305 (0.954, 5.570) | 0.0635 |
| 75-84 | 2.590 (1.381, 4.859) | **0.0030** | 5.531 (2.284, 13.395) | **0.0002** |
| 85+ | 4.102 (2.147, 7.834) | **<.0001** | 11.352 (4.573, 28.179) | **<.0001** |
| Marital Status |  |  |  |  |
| Married | 1.0 |  | 1.0 |  |
| Not Married | 1.233 (1.029, 1.477) | **0.0230** | 1.323 (1.068, 1.639) | **0.0104** |
| Preferred Language |  |  |  |  |
| English | 1.0 |  | 1.0 |  |
| Non-English | 0.999 (0.701, 1.423) | 0.9957 | 0.849 (0.539, 1.339) | 0.4828 |
| Cancer Type |  |  |  |  |
| >1 cancer type* | 0.582 (0.269, 1.260) | 0.1699 | 0.178 (0.030, 1.028) | 0.0538 |
| Brain/CNS | 1.153 (0.623, 2.133) | 0.6498 | 1.213 (0.584, 2.520) | 0.6039 |
| Breast (female) | 0.941 (0.533, 1.663) | 0.8356 | 1.899 (0.996, 3.619) | 0.0511 |
| Breast (insitu) | 0.688 (0.306, 1.544) | 0.3653 | 0.978 (0.364, 2.628) | 0.9659 |
| Colorectal | 1.884 (1.184, 2.998) | **0.0075** | 1.164 (0.706, 1.919) | 0.5493 |
| Endocrine | 0.455 (0.130, 1.594) | 0.2187 | 0.825 (0.130, 5.237) | 0.8390 |
| GI non-colorectal | 1.301 (0.833, 2.034) | 0.2468 | 1.623 (1.000, 2.633) | **0.0496** |
| Genitourinary | 0.915 (0.561, 1.491) | 0.7232 | 1.292 (0.739, 2.259) | 0.3679 |
| Gynaecological | 1.328 (0.670, 2.633) | 0.4158 | 0.785 (0.353, 1.746) | 0.5540 |
| Head & Neck | 0.730 (0.406, 1.312) | 0.2937 | 0.456 (0.213, 0.973) | **0.0425** |
| Hematologic | 1.819 (1.140, 2.901) | **0.0120** | 4.367 (2.318, 8.225) | **<.0001** |
| Lung | 0.907 (0.601, 1.369) | 0.6452 | 0.902 (0.564, 1.442) | 0.6674 |
| Melanoma | 1.043 (0.581, 1.871) | 0.8863 | 0.641 (0.289, 1.422) | 0.2741 |
| Other** | 2.120 (1.362, 3.299) | **0.0009** | 1.905 (1.179, 3.078) | **0.0084** |
| Pancreas | 5.445 (3.023, 9.807) | **<.0001** | 1.478 (0.852, 2.565) | 0.1640 |
| Prostate | 1.0 |  | 1.0 |  |
| CCI |  |  |  |  |
| 0-2 | 1.0 |  | 1.0 |  |
| 3-4 | 0.404 (0.228, 0.713) | **0.0018** | 0.322 (0.164, 0.633) | **0.0010** |
| 5+ | 0.347 (0.204, 0.590) | **<.0001** | 0.275 (0.148, 0.510) | **<.0001** |
| SEIFA |  |  |  |  |
| Most Disadvantaged | 1.0 |  | 1.0 |  |
| More disadvantaged | 1.080 (0.732, 1.594) | 0.6963 | 0.955 (0.625, 1.460) | 0.8339 |
| Average | 1.011 (0.772, 1.323) | 0.9359 | 1.038 (0.758, 1.423) | 0.8127 |
| Less disadvantaged | 0.815 (0.594, 1.119) | 0.2077 | 0.979 (0.663, 1.445) | 0.9159 |
| Least disadvantaged | 1.270 (0.565, 2.854) | 0.5625 | 0.548 (0.195, 1.535) | 0.2526 |
| MMM |  |  |  |  |
| Metropolitan | 1.0 |  | 1.0 |  |
| Regional Centres | 0.682 (0.369, 1.260) | 0.2222 | 0.553 (0.265, 1.154) | 0.1150 |
| Large rural towns | 0.573 (0.416, 0.791) | **0.0007** | 0.294 (0.198, 0.438) | **<.0001** |
| Medium rural towns | 0.364 (0.190, 0.698) | **0.0023** | 0.273 (0.132, 0.564) | **0.0005** |
| Small rural towns | 0.500 (0.275, 0.908) | **0.0228** | 0.238 (0.104, 0.540) | **0.0006** |
| Travel Time (mins) *** | *Radiotherapy facility* | | *Outpatient cancer care facility* | |
| 0-<5 | 1.0 |  | 1.0 |  |
| 5-<10 | 0.941 (0.643, 1.377) | 0.7561 | 1.186 (0.764, 1.840) | 0.4463 |
| 10-<15 | 0.790 (0.552, 1.131) | 0.1987 | 0.585 (0.384, 0.891) | **0.0126** |
| 15-<30 | 1.009 (0.714, 1.426) | 0.9574 | 0.809 (0.544, 1.205) | 0.2989 |
| 30+ | 1.269 (0.673, 2.395) | 0.4608 | 1.293 (0.615, 2.714) | 0.4971 |

Rate ratio from zero-inflated Negative Binomial regression for healthcare utilisation with count data

*’>1 Cancer type’ refers to more than 1 primary cancer site declared

**’Other’ includes all invasive cancer sites not specified above starting with ‘C’ in ICD-10 and exclude non-melanoma skin cancer

***nearest facility with health service (e.g., Emergency Department, Intensive Care Unit, Specialist Palliative Care ward)

RR= rate ratio, OR=odds ratio, CI=confidence interval, MV=mechanical ventilation
