# Supplementary material for: Does Geography Play a Role in the Receipt of End-of-Life Care for Advanced Cancer Patients? Evidence from an Australian Local Health District Population-Based Study
Source: J Palliat Med. 2023 Nov 8;26(11):1453–65. doi: 10.1089/jpm.2022.0555 (PMC10658736; doi:10.1089/jpm.2022.0555)
Supplement: Supplemental data [file Supp_TableS3.docx]

**Table S3.** Adjusted rate ratios of acute inpatient End-of-life care services and associated geographic and sociodemographic factors

| Characteristic | >1 Chemotherapy in the last 12 months |  | >1 Radiation therapy in the last 12 months |  | >1 Mechanical Ventilation in the last 12 months |  |
| --- | --- | --- | --- | --- | --- | --- |
|  | aRR (95% CI) | *P* value | aRR (95% CI) | *P* value | aRR (95% CI) | *P* value |
| Sex |  |  |  |  |  |  |
| Male | 1.0 |  | 1.0 |  | 1.0 |  |
| Female | 1.067 (0.930, 1.225) | 0.3555 | 0.970 (0.826, 1.139) | 0.7155 | 0.814 (0.676, 0.982) | **0.0313** |
| Age (years) |  |  |  |  |  |  |
| 18-44 | 1.0 |  | 1.0 |  | 1.0 |  |
| 45-54 | 1.010 (0.748, 1.362) | 0.9479 | 0.883 (0.587, 1.328) | 0.5529 | 0.648 (0.398, 1.054) | 0.0809 |
| 55-64 | 0.510 (0.382, 0.680) | **<.0001** | 0.697 (0.477, 1.018) | 0.0623 | 0.794 (0.515, 1.223) | 0.2961 |
| 65-74 | 0.311 (0.235, 0.412) | **<.0001** | 0.652 (0.450, 0.943) | **0.0233** | 0.581 (0.378, 0.891) | **0.0131** |
| 75-84 | 0.137 (0.101, 0.185) | **<.0001** | 0.376 (0.257, 0.550) | **<.0001** | 0.201 (0.127, 0.319) | **<.0001** |
| 85+ | 0.010 (0.005, 0.022) | **<.0001** | 0.168 (0.108, 0.260) | **<.0001** | 0.086 (0.049, 0.153) | **<.0001** |
| Marital Status |  |  |  |  |  |  |
| Married | 1.0 |  | 1.0 |  | 1.0 |  |
| Not Married | 0.814 (0.711, 0.933) | **0.0031** | 1.311 (1.138, 1.509) | **0.0002** | 1.032 (0.869, 1.225) | 0.7234 |
| Preferred Language |  |  |  |  |  |  |
| English | 1.0 |  | 1.0 |  | 1.0 |  |
| Non-English | 1.074 (0.813, 1.417) | 0.6135 | 0.929 (0.700, 1.234) | 0.6142 | 1.094 (1.768, 1.559) | 0.6177 |
| Cancer Type |  |  |  |  |  |  |
| >1 cancer type* | 0.768 (0.285, 2.073) | 0.6036 | 0.518 (0.305, 0.879) | **0.0149** | 2.879 (0.836, 9.912) | 0.0936 |
| Brain/CNS | 0.107 (0.028, 0.402) | **0.0009** | 0.479 (0.308, 0.746) | **0.0011** | 8.268 (3.178, 21.505) | **<.0001** |
| Breast (female) | 1.520 (0.825, 2.800) | 0.1785 | 0.378 (0.248, 0.576) | **<.0001** | 1.028 (0.295, 3.582) | 0.9647 |
| Breast (insitu) | 3.231 (1.711, 6.101) | **0.0003** | 0.406 (0.227, 0.728) | **0.0025** | 5.801 (1.954, 17.218) | **0.0015** |
| Colorectal | 0.719 (0.383, 1.351) | 0.3062 | 0.174 (0.116, 0.262) | **<.0001** | 7.267 (2.932, 18.013) | **<.0001** |
| Endocrine | 2.722 (1.147, 6.456) | **0.0230** | 0.584 (0.270, 1.263) | 0.1722 | 7.377 (2.134, 25.500) | **0.0016** |
| GI non-colorectal | 0.723 (0.399, 1.311) | 0.2864 | 0.441 (0.326, 0.597) | **<.0001** | 5.465 (2.203, 13.559) | **0.0002** |
| Genitourinary | 0.856 (0.434, 1.688) | 0.6550 | 0.582 (0.416, 0.815) | **0.0016** | 1.900 (0.651, 5.544) | 0.2398 |
| Gynaecological | 1.653 (0.829, 3.297) | 0.1532 | 0.319 (0.179, 0.566) | **<.0001** | 7.128 (2.554, 19.893) | **0.0002** |
| Head & Neck | 1.008 (0.498, 2.042) | 0.9806 | 0.609 (0.414, 0.893) | **0.0113** | 7.348 (2.847, 18.965) | **<.0001** |
| Hematologic | 9.059 (5.418, 15.145) | **<.0001** | 0.402 (0.290, 0.558) | **<.0001** | 10.684 (4.354, 26.22) | **<.0001** |
| Lung | 2.480 (1.475, 4.170) | **0.0006** | 0.576 (0.442, 0.749) | **<.0001** | 3.562 (1.438, 8.818) | **0.0060** |
| Melanoma | 1.350 (0.698, 2.609) | 0.3719 | 0.541 (0.370, 0.791) | **0.0015** | 7.939 (3.082, 20.452) | **<.0001** |
| Other** | 1.398 (0.799, 2.446) | 0.2397 | 0.226 (0.157, 0.323) | **<.0001** | 5.935 (2.399, 14.686) | **0.0001** |
| Pancreas | 1.714 (0.963, 3.051) | 0.0667 | 0.094 (0.052, 0.171) | **<.0001** | 4.403 (1.709, 11.341) | **0.0021** |
| Prostate | 1.0 |  | 1.0 |  | 1.0 |  |
| CCI |  |  |  |  |  |  |
| 0-2 | 1.0 |  | 1.0 |  | 1.0 |  |
| 3-4 | 2.020 (1.289, 3.168) | **0.0022** | 1.306 (0.778, 2.191) | 0.3116 | 0.398 (0.271, 0.585) | **<.0001** |
| 5+ | 1.461 (0.938, 2.275) | 0.0933 | 1.865 (1.155, 3.012) | **0.0107** | 0.434 (0.309, 0.610) | **<.0001** |
| SEIFA |  |  |  |  |  |  |
| Most Disadvantaged | 1.0 |  | 1.0 |  | 1.0 |  |
| More disadvantaged | 1.022 (0.778, 1.341) | 0.8737 | 1.273 (0.936, 1.731) | 0.1229 | 0.634 (0.434, 0.925) | **0.0182** |
| Average | 0.996 (0.821, 1.208) | 0.9727 | 0.897 (0.737, 1.091) | 0.2776 | 0.842 (0.654, 1.085) | 0.1850 |
| Less disadvantaged | 1.102 (0.886, 1.370) | 0.3800 | 1.001 (0.798, 1.257) | 0.9869 | 0.644 (0.466, 0.891) | **0.0079** |
| Least disadvantaged | 0.967 (0.542, 1.723) | 0.9095 | 1.024 (0.614, 1.707) | 0.9252 | 0.910 (0.467, 1.772) | 0.7824 |
| MMM |  |  |  |  |  |  |
| Metropolitan | 1.0 |  | 1.0 |  | 1.0 |  |
| Regional Centres | 1.208 (0.824, 1.770) | 0.3325 | 0.534 (0.303, 0.942) | **0.0305** | 2.198 (1.299, 3.719) | **0.0033** |
| Large rural towns | 0.915 (0.730, 1.146) | 0.4397 | 0.320 (0.231, 0.444) | **<.0001** | 0.951 (0.697, 1.298) | 0.7536 |
| Medium rural towns | 0.890 (0.568, 1.394) | 0.6121 | 0.367 (0.193, 0.699) | **0.0023** | 1.695 (0.985, 2.917) | 0.0563 |
| Small rural towns | 0.669 (0.435, 1.029) | 0.0674 | 0.071 (0.028, 0.180) | **<.0001** | 2.020 (1.211, 3.368) | **0.0070** |
| Travel Time (mins) *** | *Chemotherapy facility* | | *Radiation Oncology facility* | | *MV facility* | |
| 0-<5 | 1.0 |  | 1.0 |  | 1.0 |  |
| 5-<10 | 0.969 (0.740, 1.268) | 0.8208 | 0.737 (0.556, 0.975) | **0.0331** | 0.831 (0.590, 1.169) | 0.2880 |
| 10-<15 | 0.892 (0.684, 1.164) | 0.4021 | 0.914 (0.710, 1.176) | 0.4862 | 0.708 (0.501, 1.001) | 0.0508 |
| 15-<30 | 1.186 (0.931, 1.510) | 0.1658 | 1.009 (0.793, 1.285) | 0.9371 | 0.834 (0.605, 1.151) | 0.2710 |
| 30+ | 0.853 (0.540, 1.348) | 0.4973 | 0.956 (0.516, 1.773) | 0.8889 | 0.580 (0.337, 1.000) | 0.0503 |

Rate ratio from Negative Binomial regression for healthcare utilisation with count data and odds ratio from binary acute end-of-life care service from logistic regression of binary acute end-of-life care service utilisation data

*’>1 Cancer type’ refers to more than 1 primary cancer site declared

**’Other’ includes all invasive cancer sites not specified above starting with ‘C’ in ICD-10 and exclude non-melanoma skin cancer

***nearest facility with health service (e.g., Emergency Department, Intensive Care Unit, Specialist Palliative Care ward)

RR= rate ratio, OR=odds ratio, CI=confidence interval, MV=mechanical ventilation
