# Supplementary material for: Does Geography Play a Role in the Receipt of End-of-Life Care for Advanced Cancer Patients? Evidence from an Australian Local Health District Population-Based Study
Source: J Palliat Med. 2023 Nov 8;26(11):1453–65. doi: 10.1089/jpm.2022.0555 (PMC10658736; doi:10.1089/jpm.2022.0555)
Supplement: Supplemental data [file Supp_TableS4.docx]

**Table S4.** Adjusted rate ratios of in-patient Palliative End-of-life care services and associated geographic and sociodemographic factors

| Characteristic | Stays receiving PC in the last 12 months of life | | Stays receiving PC in the last 12 months of life (prior to last stay) | |
| --- | --- | --- | --- | --- |
|  | aRR (95% CI) | *P* value | aRR (95% CI) | *P* value |
| Sex |  |  |  |  |
| Male | 1.0 |  | 1.0 |  |
| Female | 1.020 (0.980, 1.061) | 0.3291 | 1.015 (0.948, 1.087) | 0.6549 |
| Age (years) |  |  |  |  |
| 18-44 | 1.0 |  | 1.0 |  |
| 45-54 | 0.945 (0.829, 1.077) | 0.4022 | 0.938 (0.759, 1.159) | 0.5581 |
| 55-64 | 0.830 (0.735, 0.937) | **0.0027** | 0.769 (0.631, 0.936) | **0.0090** |
| 65-74 | 0.770 (0.684, 0.867) | **<.0001** | 0.686 (0.566, 0.831) | **0.0001** |
| 75-84 | 0.693 (0.616, 0.781) | **<.0001** | 0.582 (0.480, 0.706) | **<.0001** |
| 85+ | 0.585 (0.517, 0.662) | **<.0001** | 0.433 (0.353, 0.530) | **<.0001** |
| Marital Status |  |  |  |  |
| Married | 1.0 |  | 1.0 |  |
| Not Married | 0.944 (0.910, 0.980) | **0.0024** | 0.919 (0.863, 0.979) | **0.0093** |
| Preferred Language |  |  |  |  |
| English | 1.0 |  | 1.0 |  |
| Non-English | 1.037 (0.967, 1.113) | 0.3005 | 1.062 (0.942, 1.198) | 0.3189 |
| Cancer Type |  |  |  |  |
| >1 cancer type* | 0.971 (0.833, 1.132) | 0.7112 | 0.930 (0.719, 1.203) | 0.5826 |
| Brain/CNS | 0.739 (0.644, 0.848) | **<.0001** | 0.580 (0.455, 0.738) | **<.0001** |
| Breast (female) | 0.805 (0.715, 0.907) | **0.0004** | 0.715 (0.584, 0.876) | **0.0012** |
| Breast (insitu) | 0.809 (0.684, 0.957) | **0.0134** | 0.719 (0.540, 0.957) | **0.0240** |
| Colorectal | 0.855 (0.779, 0.939) | **0.0011** | 0.777 (0.662, 0.910) | **0.0018** |
| Endocrine | 0.837 (0.648, 1.079) | 0.1711 | 0.731 (0.470, 1.137) | 0.1648 |
| GI non-colorectal | 0.891 (0.813, 0.975) | **0.0129** | 0.816 (0.700, 0.951) | **0.0095** |
| Genitourinary | 0.904 (0.817, 1.001) | 0.0527 | 0.897 (0.757, 1.063) | 0.2100 |
| Gynaecological | 0.854 (0.741, 0.985) | **0.0312** | 0.772 (0.604, 0.986) | **0.0388** |
| Head & Neck | 0.806 (0.708, 0.918) | **0.0012** | 0.719 (0.576, 0.897) | **0.0036** |
| Hematologic | 1.008 (0.921, 1.102) | 0.8585 | 0.988 (0.851, 1.148) | 0.8840 |
| Lung | 0.895 (0.823, 0.974) | **0.0102** | 0.830 (0.721, 0.956) | **0.0101** |
| Melanoma | 0.778 (0.686, 0.881) | **<.0001** | 0.680 (0.548, 0.844) | **0.0005** |
| Other** | 0.830 (0.759, 0.908) | **<.0001** | 0.754 (0.647, 0.878) | **0.0003** |
| Pancreas | 0.881 (0.795, 0.976) | **0.0160** | 0.801 (0.674, 0.953) | **0.0127** |
| Prostate | 1.0 |  | 1.0 |  |
| CCI |  |  |  |  |
| 0-2 | 1.0 |  | 1.0 |  |
| 3-4 | 1.158 (1.045, 1.283) | **0.0049** | 1.068 (0.901, 1.267) | 0.4434 |
| 5+ | 1.181 (1.074, 1.298) | **0.0006** | 1.043 (0.891, 1.220) | 0.5963 |
| SEIFA |  |  |  |  |
| Most Disadvantaged | 1.0 |  | 1.0 |  |
| More disadvantaged | 0.977 (0.902, 1.059) | 0.5869 | 0.970 (0.845, 1.113) | 0.6662 |
| Average | 0.999 (0.945, 1.055) | 0.9726 | 0.985 (0.898, 1.081) | 0.7632 |
| Less disadvantaged | 0.996 (0.933, 1.065) | 0.9273 | 0.985 (0.898, 1.081) | 0.6912 |
| Least disadvantaged | 1.051 (0.888, 1.244) | 0.5585 | 1.039 (0.785, 1.377) | 0.7843 |
| MMM |  |  |  |  |
| Metropolitan | 1.0 |  | 1.0 |  |
| Regional Centres | 0.851 (0.746, 0.971) | **0.0171** | 0.754 (0.600, 0.947) | **0.0152** |
| Large rural towns | 0.959 (0.896, 1.026) | 0.2305 | 0.940 (0.837, 1.056) | 0.3009 |
| Medium rural towns | 1.008 (0.868, 1.171) | 0.9102 | 0.984 (0.760, 1.274) | 0.9060 |
| Small rural towns | 0.892 (0.780, 1.021) | 0.0978 | 0.825 (0.655, 1.038) | 0.1013 |
| Travel Time (mins) *** | *PC facility* |  | *PC facility* | |
| 0-<5 | 1.0 |  | 1.0 |  |
| 5-<10 | 1.000 (0.933, 1.071) | 0.9980 | 0.986 (0.877, 1.110) | 0.8270 |
| 10-<15 | 1.038 (0.982, 1.098) | 0.1840 | 1.056 (0.961, 1.161) | 0.2518 |
| 15-<30 | 1.045 (0.961, 1.135) | 0.2982 | 1.105 (0.962, 1.270) | 0.1567 |
| 30+ | 0.965 (0.828, 1.126) | 0.6578 | 0.949 (0.728, 1.237) | 0.7001 |

Rate ratio from Poisson regression for end-of-life care service utilisation

*’>1 Cancer type’ refers to more than 1 primary cancer site declared

**’Other’ includes all invasive cancer sites not specified above starting with ‘C’ in ICD-10 & exclude non-melanoma skin cancer

***nearest facility with health service (e.g., Emergency Department, Intensive Care Unit, Specialist Palliative Care ward)

RR= rate ratio, OR=odds ratio, CI=confidence interval, MV=mechanical ventilation
