# Supplementary material for: Does Geography Play a Role in the Receipt of End-of-Life Care for Advanced Cancer Patients? Evidence from an Australian Local Health District Population-Based Study
Source: J Palliat Med. 2023 Nov 8;26(11):1453–65. doi: 10.1089/jpm.2022.0555 (PMC10658736; doi:10.1089/jpm.2022.0555)
Supplement: Supplemental data [file Supp_TableS5.docx]

**Table S5.** Adjusted rate ratios of in-patient Specialist Palliative End-of-life care services and associated geographic and sociodemographic factors

| Characteristic | Stays receiving SPC in the last 12 months of life | | Stays receiving SPC in the last 12 months of life (prior to last stay) | |
| --- | --- | --- | --- | --- |
|  | aRR (95% CI) | *P* value | aRR (95% CI) | *P* value |
| Sex |  |  |  |  |
| Male | 1.0 |  | 1.0 |  |
| Female | 1.140 (1.051, 1.237) | **0.0015** | 1.217 (1.024, 1.447) | **0.0253** |
| Age (years) |  |  |  |  |
| 18-44 | 1.0 |  | 1.0 |  |
| 45-54 | 0.876 (0.668, 1.149) | 0.3412 | 0.852 (0.528, 1.373) | 0.5109 |
| 55-64 | 0.921 (0.720, 1.179) | 0.5161 | 0.835 (0.540, 1.290) | 0.4176 |
| 65-74 | 0.853 (0.670, 1.086) | 0.1988 | 0.637 (0.414, 0.978) | **0.0395** |
| 75-84 | 0.823 (0.646, 1.048) | 0.1152 | 0.598 (0.388, 0.921) | **0.0199** |
| 85+ | 0.725 (0.565, 0.931) | **0.0119** | 0.339 (0.211, 0.545) | **<.0001** |
| Marital Status |  |  |  |  |
| Married | 1.0 |  | 1.0 |  |
| Not Married | 0.932 (0.865, 1.004) | 0.0664 | 0.694 (0.593, 0.813) | **<.0001** |
| Preferred Language |  |  |  |  |
| English | 1.0 |  | 1.0 |  |
| Non-English | 0.870 (0.749, 1.010) | 0.0681 | 0.962 (0.698, 1.328) | 0.8179 |
| Cancer Type |  |  |  |  |
| >1 cancer type* | 0.855 (0.625, 1.169) | 0.3278 | 0.247 (0.108, 0.564) | **0.0009** |
| Brain/CNS | 0.899 (0.695, 1.163) | 0.4211 | 0.537 (0.334, 0.862) | **0.0101** |
| Breast (female) | 0.890 (0.709, 1.117) | 0.3169 | 0.550 (0.363, 0.834) | **0.005** |
| Breast (insitu) | 1.057 (0.788, 1.417) | 0.7106 | 0.946 (0.585, 1.530) | 0.8234 |
| Colorectal | 0.858 (0.713, 1.033) | 0.1075 | 0.554 (0.400, 0.767) | **0.0004** |
| Endocrine | 0.886 (0.545, 1.440) | 0.6264 | 0.251 (0.062, 1.007) | 0.0513 |
| GI non-colorectal | 0.860 (0.717, 1.031) | 0.1037 | 0.398 (0.284, 0.559) | **<.0001** |
| Genitourinary | 0.824 (0.672, 1.011) | 0.0645 | 0.491 (0.336, 0.718) | **0.0002** |
| Gynaecological | 0.948 (0.727, 1.237) | 0.6992 | 0.722 (0.458, 1.138) | 0.1615 |
| Head & Neck | 0.785 (0.603, 1.023) | 0.0737 | 0.246 (0.127, 0.474) | **<.0001** |
| Hematologic | 0.681 (0.563, 0.824) | **<.0001** | 0.264 (0.178, 0.390) | **<.0001** |
| Lung | 0.800 (0.676, 0.947) | **0.0098** | 0.470 (0.351, 0.630) | **<.0001** |
| Melanoma | 0.743 (0.578, 0.955) | **0.0206** | 0.350 (0.209, 0.585) | **<.0001** |
| Other** | 0.771 (0.644, 0.923) | **0.0048** | 0.336 (0.237, 0.477) | **<.0001** |
| Pancreas | 0.940 (0.771, 1.147) | 0.5462 | 0.508 (0.351, 0.736) | **0.0003** |
| Prostate | 1.0 |  | 1.0 |  |
| CCI |  |  |  |  |
| 0-2 | 1.0 |  | 1.0 |  |
| 3-4 | 2.453 (1.850, 3.253) | **<.0001** | 4.111 (1.808, 9.343) | **0.0007** |
| 5+ | 2.552 (1.948, 3.342) | **<.0001** | 4.456 (2.008, 9.889) | **0.0002** |
| SEIFA |  |  |  |  |
| Most Disadvantaged | 1.0 |  | 1.0 |  |
| More disadvantaged | 1.173 (0.992, 1.388) | 0.0616 | 1.391 (0.992, 1.951) | 0.0554 |
| Average | 1.079 (0.971, 1.199) | 0.1546 | 1.081 (0.863, 1.353) | 0.4957 |
| Less disadvantaged | 0.978 (0.857, 1.116) | 0.7461 | 0.917 (0.694, 1.212) | 0.5449 |
| Least disadvantaged | 1.188 (0.815, 1.588) | 0.4457 | 1.395 (0.764, 2.512) | 0.2823 |
| MMM |  |  |  |  |
| Metropolitan | 1.0 |  | 1.0 |  |
| Regional Centres | 1.114 (0.866, 1.433) | 0.3966 | 0.853 (0.506, 1.437) | 0.5515 |
| Large rural towns | 1.108 (0.950, 1.292) | 0.1911 | 1.157 (0.859, 1.558) | 0.3351 |
| Medium rural towns | 0.924 (0.616, 1.386) | 0.7039 | 1.091 (0.535, 2.224) | 0.8087 |
| Small rural towns | 1.098 (0.702, 1.717) | 0.6817 | 0.759 (0.326, 1.765) | 0.5221 |
| Travel Time (mins) *** | *SPC facility* | | *SPC facility* | |
| 0-<5 | 1.0 |  | 1.0 |  |
| 5-<10 | 0.875 (0.752, 1.018) | 0.0860 | 0.742 (0.522, 1.054) | 0.0958 |
| 10-<15 | 0.996 (0.891, 1.114) | 0.9563 | 1.276 (1.001, 1.626) | **0.0484** |
| 15-<30 | 0.957 (0.823, 1.112) | 0.5707 | 1.477 (1.098, 1.986) | **0.0098** |
| 30+ | 0.723 (0.476, 1.098) | 0.1286 | 0.761 (0.357, 1.623) | 0.4803 |

Rate ratio from Poisson regression for end-of-life care service utilisation

*’>1 Cancer type’ refers to more than 1 primary cancer site declared

**’Other’ includes all invasive cancer sites not specified above starting with ‘C’ in ICD-10 & exclude non-melanoma skin cancer

***nearest facility with health service (e.g., Emergency Department, Intensive Care Unit, Specialist Palliative Care ward)

RR= rate ratio, OR=odds ratio, CI=confidence interval, MV=mechanical ventilation
