# Supplementary material for: Does Geography Play a Role in the Receipt of End-of-Life Care for Advanced Cancer Patients? Evidence from an Australian Local Health District Population-Based Study
Source: J Palliat Med. 2023 Nov 8;26(11):1453–65. doi: 10.1089/jpm.2022.0555 (PMC10658736; doi:10.1089/jpm.2022.0555)
Supplement: Supplemental data [file Supp_TableS6.docx]

**Table S6.** Adjusted rate ratios of outpatient Specialist Palliative Care and Chemotherapy services and associated geographic and sociodemographic factors

| Characteristic | Receipt of Outpatient  SPC services | | Receipt of Outpatient Chemotherapy treatment | |
| --- | --- | --- | --- | --- |
|  | **Zero-inflation model** | | **Zero-inflation model** | |
|  | aRR (95% CI) | *P* value | aRR (95% CI) | *P* value |
| Intercept | 0.968 |  | 2.384 |  |
| Sex |  |  |  |  |
| Male | 1.0 |  | 1.0 |  |
| Female | 0.751 (0.601, 0.937) | **0.0114** | 0.872 (0.710, 1.072) | 0.1953 |
| Age (years) |  |  |  |  |
| 18-44 | 1.0 |  | 1.0 |  |
| 45-54 | 1.052 (0.503, 2.197) | 0.8925 | 1.122 (0.549, 2.293) | 0.7517 |
| 55-64 | 1.072 (0.551, 2.085) | 0.8369 | 1.501 (0.783, 2.878) | 0.2204 |
| 65-74 | 1.402 (0.734, 2.681) | 0.3057 | 2.222 (1.175, 4.201) | **0.0140** |
| 75-84 | 1.593 (0.837, 3.031) | 0.1558 | 4.220 (2.226, 8.002) | **<.0001** |
| 85+ | 1.763 (0.906, 3.431) | 0.0947 | 14.306 (7.211, 28.377) | **<.0001** |
| Marital Status |  |  |  |  |
| Married | 1.0 |  | 1.0 |  |
| Not Married | 1.586 (1.287, 1.954) | **<.0001** | 1.226 (1.014, 1.482) | **0.0346** |
| Preferred Language |  |  |  |  |
| English | 1.0 |  | 1.0 |  |
| Non-English | 0.819 (0.535, 1.254) | 0.3594 | 1.180 (0.824, 1.690) | 0.3651 |
| Cancer Type |  |  |  |  |
| >1 cancer type* | 0.421 (0.069, 2.557) | 0.3479 | 1.233 (0.551, 2.759) | 0.6096 |
| Brain/CNS | 3.222 (1.585, 6.550) | **0.0012** | 4.029 (1.848, 8.782) | **0.0005** |
| Breast (female) | 1.661 (0.831, 3.320) | 0.1506 | 1.577 (0.854, 2.911) | 0.1449 |
| Breast (insitu) | 0.391 (0.071, 2.132) | 0.2781 | 1.784 (0.797, 3.994) | 0.1589 |
| Colorectal | 1.483 (0.829, 2.651) | 0.1833 | 1.043 (0.644, 1.689) | 0.8634 |
| Endocrine | 4.208 (1.073, 16.497) | **0.0392** | 1.329 (0.300, 5.887) | 0.7074 |
| GI non-colorectal | 1.670 (0.949, 2.939) | 0.0752 | 1.962 (1.195, 3.221) | **0.0076** |
| Genitourinary | 1.951 (1.072, 3.55) | **0.0286** | 1.288 (0.733, 2.262) | 0.3784 |
| Gynaecological | 1.144 (0.453, 2.89) | 0.7746 | 1.003 (0.488, 2.060) | 0.9934 |
| Head & Neck | 1.964 (0.988, 3.902) | 0.0539 | 1.124 (0.576, 2.192) | 0.7313 |
| Hematologic | 5.189 (2.964, 9.082) | **<.0001** | 0.902 (0.561, 1.449) | 0.6703 |
| Lung | 1.788 (1.063, 3.007) | **0.0284** | 1.147 (0.736, 1.789) | 0.5434 |
| Melanoma | 2.064 (1.018, 4.183) | **0.0443** | 1.039 (0.550, 1.964) | 0.9043 |
| Other** | 2.607 (1.526, 4.454) | **0.0005** | 1.561 (0.974, 2.499) | 0.0637 |
| Pancreas | 1.070 (0.548, 2.087) | 0.8426 | 1.317 (0.780, 2.221) | 0.3016 |
| Prostate | 1.0 |  | 1.0 |  |
| CCI |  |  |  |  |
| 0-2 | 1.0 |  | 1.0 |  |
| 3-4 | 0.256 (0.150, 0.438) | **<.0001** | 0.415 (0.236, 0.729) | **0.0022** |
| 5+ | 0.295 (0.182, 0.479) | **<.0001** | 0.315 (0.186, 0.532) | **<.0001** |
| SEIFA |  |  |  |  |
| Most Disadvantaged | 1.0 |  | 1.0 |  |
| More disadvantaged | 0.623 (0.386, 1.005) | 0.0528 | 0.990 (0.666, 1.472) | 0.9628 |
| Average | 1.002 (0.749, 1.341) | 0.9874 | 0.900 (0.689, 1.174) | 0.4380 |
| Less disadvantaged | 1.026 (0.712, 1.479) | 0.8871 | 1.191 (0.865, 1.640) | 0.2816 |
| Least disadvantaged | 1.022 (0.388, 2.691) | 0.9646 | 0.719 (0.334, 1.547) | 0.3998 |
| MMM |  |  |  |  |
| Metropolitan | 1.0 |  | 1.0 |  |
| Regional Centres | 0.937 (0.412, 2.128) | 0.8771 | 0.584 (0.319, 1.070) | 0.0819 |
| Large rural towns | 0.587 (0.402, 0.857) | **0.0058** | 0.166 (0.114, 0.240) | **<.0001** |
| Medium rural towns | 1.086 (0.492, 2.400) | 0.8367 | 0.121 (0.059, 0.246) | **<.0001** |
| Small rural towns | 1.972 (0.936, 4.154) | 0.0740 | 0.185 (0.094, 0.364) | **<.0001** |
| Travel Time (mins) *** | *SPC facility* | | *Chemotherapy facility* | |
| 0-<5 | 1.0 |  | 1.0 |  |
| 5-<10 | 0.855 (0.596, 1.227) | 0.3970 | 0.873 (0.595, 1.280) | 0.4873 |
| 10-<15 | 0.843 (0.622, 1.142) | 0.2723 | 0.603 (0.419, 0.867) | **0.0064** |
| 15-<30 | 0.584 (0.360, 0.947) | **0.0293** | 0.724 (0.508, 1.034) | 0.0760 |
| 30+ | 0.840 (0.370, 1.909) | 0.6781 | 1.027 (0.526, 2.004) | 0.9376 |

Rate ratio from zero-inflated Negative Binomial regression for healthcare utilisation with count data

*’>1 Cancer type’ refers to more than 1 primary cancer site declared

**’Other’ includes all invasive cancer sites not specified above starting with ‘C’ in ICD-10 and exclude non-melanoma skin cancer

***nearest facility with health service (e.g., Emergency Department, Intensive Care Unit, Specialist Palliative Care ward)

RR= rate ratio, OR=odds ratio, CI=confidence interval, MV=mechanical ventilation
